# Academic Medicine's Responses to National Race-Related Events and Its Role in Civil Rights

**DOI:** 10.1089/heq.2017.0052

**Published:** 2018-03-01

**Authors:** Paul B. Perrin

**Affiliations:** Department of Psychology, Virginia Commonwealth University, Richmond, Virginia.

These past few years have been difficult for our nation, as well for many of our patients and for those of us who treat, conduct research with, and teach about health issues affecting racial/ethnic minority populations. The national racial climate has channeled directly into our professional work, and indeed into many of our lives. To many, the list of names of black men killed has felt endless: Keith Lamont Scott, Terence Crutcher, Freddie Gray, Michael Brown, Alton Sterling, among far too many others. People have taken to the streets protesting these killings, and a Black Lives Matter movement has gained substantial momentum, contributing to what some have called the Second Civil Rights Movement.

Some of us in the medical community feel that race relations in the United States have reached the lowest point in our lifetimes, even some of us who remember segregation. Many patients of color come into our clinics, offices, and research studies reeling from these national race-related events, and the political rhetoric and related public policies that disproportionately harm immigrants and communities of color seem only to make that pain worse. Our trainees and colleagues of color, as well as white allies, struggle to make sense of this era of highly visible racism. Although many people believed that racism had gone undercover, there is no doubt now that it has reemerged: it is alive, pervasive, and blatant.

This overt racism, now combining synergistically with more covert forms, has taken a profound mental and physical health toll on many of us and the populations we serve. Universities and training programs in the allied health professions across the nation have sent supportive emails over listservs, held antiracism and cultural competence trainings, hosted racial dialogues, and sponsored guest speakers. Despite these efforts, the pain of racism is not fading. And why would it? To this very day, my trainees of color leave my office in Richmond, Virginia, for their rotations at our community clinic and in four blocks turn right from Monument Avenue under a statue of J.E.B. Stuart. If they strolled another block, they would pass beneath a monument to Robert E. Lee atop his horse, adorning Richmond's largest roundabout. Racism is far from over, and we are becoming increasingly aware of its role in medicine.

Exactly 50 years ago in September 1967, Dr. Martin Luther King, Jr. gave a speech to a large group of behavioral health researchers in Washington, DC. The speech was published in March 1968,^1^ the month before his assassination. Dr. King told us that health scientists have a unique role to play in the Civil Rights Movement, and I find it both helpful and troubling that his words ring as true today as they did in 1967. I look to his words, now 50 years old, for perspective.

He understood many of our feelings, telling us, “I must confess that these have been very difficult days for me personally. And these have been difficult days for every Civil Rights leader, for every lover of justice and peace. They have been days of frustration—days when we could not quite see where we were going, and when we often felt that our works were in vain, days when we were tempted to end up in the valley of despair” (p. 185). Like him, not only our patients but—I would argue—the medical community itself is often tempted to despair, and we want the race-related pain that we and our patients feel to stop, particularly those of us of color.

But Dr. King cautioned us about our pain: “Men and women should be as maladjusted as the prophet Amos, who in the midst of the injustices of his day, could cry out in words that echo across the centuries, ‘Let justice roll down like waters and righteousness like a mighty stream’” (p. 185). He challenged us not to acclimate to racial injustice, as much as we might be tempted to self-soothe and withdraw or encourage our patients to. He reminded us that the medical field gave society the great word “maladjusted,” but he implored us to “never adjust ourselves to racial discrimination and racial segregation” (p. 185). He told us our pain was purposeful and necessary, and though we often grow weary, we should not allow it to be numbed or quieted. Without it, we would lack the fuel and vigor we need to assume our unique role in the Civil Rights Movement.

Never has the medical community been more capable of our assuming the role he described; never has our scientific understanding of the health effects of racism been more nuanced and comprehensive. For our patients of color, we must begin to shift our approach from solely remediating the physical and mental health effects of racism to preventing them. We cannot simply numb the symptoms of immigrant patients presenting in our offices with insomnia when the true source of their pathology is fear of deportation. We similarly cannot fully treat post-traumatic stress disorder stemming from racial trauma unless we leave our offices to perform antiracism community work, engaging in prevention before we encounter patients of color in a medical capacity. We cannot stand by and solely treat hypertension in patients of color without taking stances on civil rights legislation that our research shows would directly influence their stress and health.

There are several concrete steps that academic health centers can take to draw on our civil rights mission in the midst of national race-related events. At the individual level, health faculty and healthcare providers can directly ask both trainees and patients of color about their emotions related to these events, and then overtly validate those emotions, being respectful of the profound range of reactions people may have. Doing so can build support into medical and training environments that otherwise tend to emphasize more objective and sometimes sterile approaches. It is also important that we challenge ourselves to reflect on and consider the various ways in which we may unwittingly reinforce racism in our profession through the unconscious biases that we all have. Ultimately, it is on us to educate ourselves about the privileges we hold based on aspects of our identities or social status and how those privileges may blind us to the ways that racism can become embedded in our training environments and clinics. At the macro level, we can join forces and advocate in local, state, and federal contexts for our patients of color and help provide the civil rights that will positively influence their health. The American Medical Association and many other allied health professional organizations have grassroots advocacy and patient action networks through which health professionals can get involved in legislative work to improve access to care for patients of color.

If ever a time for the medicine to assume its role, it is now. This is not the time for medical professionals—no matter exhausted themselves—to go quietly into the night. We must bend our pain to action and as Dr. King has said, “through such creative maladjustment, we may be able to emerge from the bleak and desolate midnight of man's inhumanity to man, into the bright and glittering daybreak of freedom and justice” (p. 185).
